# Hsp90 Inhibitors for the Treatment of Chronic Myeloid Leukemia

**DOI:** 10.1155/2015/757694

**Published:** 2015-12-03

**Authors:** Kalubai Vari Khajapeer, Rajasekaran Baskaran

**Affiliations:** Department of Biochemistry and Molecular Biology, School of Life Sciences, Pondicherry University, Pondicherry 605014, India

## Abstract

Chronic myeloid leukemia (CML) is a hematological malignancy that arises due to reciprocal translocation of 3′ sequences from c-Abelson (ABL) protooncogene of chromosome 9 with 5′ sequence of truncated break point cluster region (BCR) on chromosome 22. BCR-ABL is a functional oncoprotein p210 that exhibits constitutively activated tyrosine kinase causing genomic alteration of hematopoietic stem cells. BCR-ABL specific tyrosine kinase inhibitors (TKIs) successfully block CML progression. However, drug resistance owing to BCR-ABL mutations and overexpression is still an issue. Heat-shock proteins (Hsps) function as molecular chaperones facilitating proper folding of nascent polypeptides. Their increased expression under stressful conditions protects cells by stabilizing unfolded or misfolded peptides. Hsp90 is the major mammalian protein and is required by BCR-ABL for stabilization and maturation. Hsp90 inhibitors destabilize the binding of BCR-ABL protein thus leading to the formation of heteroprotein complex that is eventually degraded by the ubiquitin-proteasome pathway. Results of many novel Hsp90 inhibitors that have entered into various clinical trials are encouraging. The present review targets the current development in the CML treatment by availing Hsp90 specific inhibitors.

## 1. Introduction

Leukemia is a type of blood cancer in which unusual raise in number of white blood cells is found. Four types of leukemia are recognized by most cancer registries [1]. They are acute myeloid leukemia (AML), acute lymphoid leukemia (ALL), chronic myeloid leukemia (CML), and chronic lymphoid leukemia (CLL). CML is a hematoproliferative neoplasm that is marked by uncontrolled myeloid cell divisions in bone marrow [2]. CML is divided into three phases—chronic phase, accelerated phase, and blast crisis [3]. Although found in all age groups, CML more often occurs in middle aged and elderly people with a median age of 67 years [4]. CML accounts for ~20% of all leukemia cases in adults in western population [5]. Estimated annual prevalence of CML is 1-2 per 1,00,000 population with greater frequency in males [6].

The mark of CML is the presence of shortened Philadelphia chromosome (Ph) that occurs due to reciprocal translocation between chromosome 9 and chromosome 22 [(9;22) (q34;q11)], thereby eventually culminating in the genesis of the BCR-ABL oncogene. About 90% of CML patients have Ph. The BCR-ABL oncogene encodes a constitutively activated tyrosine kinase, BCR-ABL. Fusion at different break points in BCR gene locus produces 3 different oncoproteins, namely, p190, p210, and p230. p190 causes ALL [7]; p210 is the major protein involved in CML [2]. p230 is correlated with a mild form of CML.

## 2. Signaling Pathways Affected by BCR-ABL

**Figure 1:**
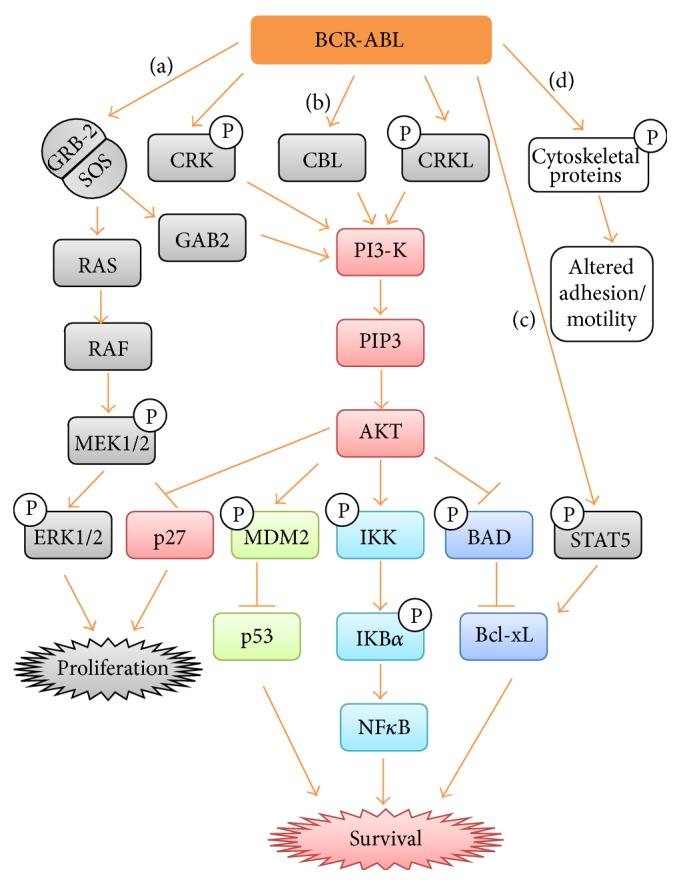
Signaling pathways activated by BCR-ABL. (a) BCR-ABL activates GRB-2/SOS which in turn activates RAS. Active RAS activates RAF. Active RAF stimulates MEK1, which in turn activates ERK1/2. Activation of Ras pathway by BCR-ABL aids CML cells proliferation. On the other hand, activated GRB-2/SOS stimulates GAB2 which activates PI3-K pathway. (b) BCR-ABL phosphorylates adaptor proteins like CRK and CRKL leading to the activation of PI3-K. PI3-K phosphorylates PIP2 to PIP3 which in turn activates AKT. AKT inhibits p27 leading to CML cells proliferation. AKT phosphorylates MDM2, which in turn inhibit p53. AKT activates NF*κ*B via phosphorylation of IKK and IkB*α*. AKT inhibits p-BAD. Activation of NF*κ*B and inhibition of p53 and BAD by AKT evade apoptosis and promote CML cells survival. (c) BCR-ABL phosphorylates STAT5 which also aid in evading apoptosis of CML cells. (d) BCR-ABL phosphorylate cytoskeleton proteins resulting in increased cellular motility and reduced adhesion to extracellular matrix of bone marrow.

BCR-ABL activates several pathways such as RAS, a small GTPase, mitogen activated protein kinase (MAPK), signal transducers and activator of transcription (STAT), and phosphoinositide 3-kinase (PI3K) pathways that regulate survival, proliferation, and apoptosis of leukemic cells [8–16] (Figure 1).

## 3. Overview of Treatment for CML

**Figure 2:**
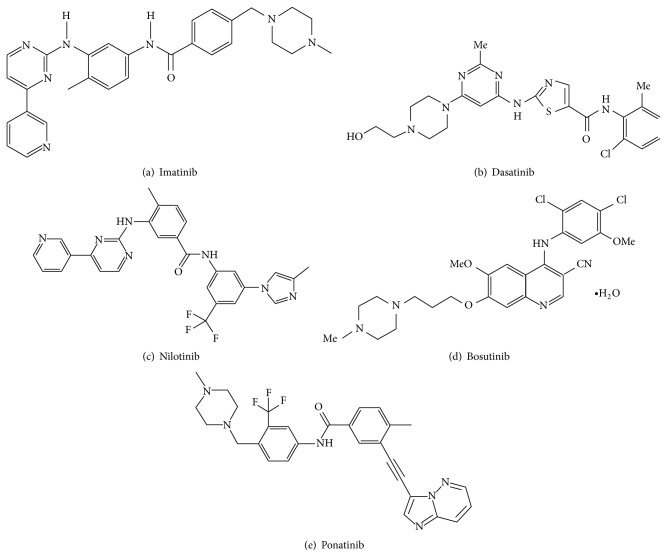
Currently available BCR-ABL specific TKIs for CML treatment. (a) First-generation TKI. ((b), (c), and (d)) Second-generation TKIs. (e) Third-generation TKI.

Since 1960, antimetabolites like cytarabine, busulfan (BUS), and hydroxyurea (HU) were used for the treatment of CML [8]. Later in 1970, allogeneic stem cell transplantation, in which patient receives stem cells from a genetically similar donor, was used as an effective therapy for CML. But this approach was risky due to limited availability of genetically identical persons and caused death in elderly people [17]. In 1980, interferon-*α* (IFN-*α*) proved effective for the treatment of CML. Such treatment prolonged the survival compared to BUS and HU [18]. Later, the discovery of tyrosine kinase inhibitors (TKIs) renewed the CML treatment (Figure 2) with increased survival rates, decreased side effects, and improved life quality (Table 1).

### 3.1. First-Generation TKI

Imatinib, a 2-phenylaminopyrimidine derivative (Figure 2), competes with the ATP binding site of c-ABL kinase of fusion BCR-ABL, found in CML cells [19]. Phase-I trials of imatinib showed significant antiproliferative activity in CML patients in which IFN-*α* treatment failed [20]. In addition to ABL, imatinib inhibits PDGFR, Arg, and c-Kit except Src kinases. Results of International Randomized Study of Interferon and STI571 (IRIS) trials showed reliability and supremacy of imatinib related to IFN-*α* in terms of hematologic and cytogenetic responses [21]. Imatinib was legalized by the US, Food and Drug Administration (FDA) in 2001.

### 3.2. Resistance of Imatinib

Resistance of imatinib is of two types, BCR-ABL dependent and BCR-ABL independent. The former is due to mutations in the BCR-ABL kinase domain [22] and overexpression of BCR-ABL protein [23]. Point mutations in the BCR-ABL kinase domain decrease or inhibit the interaction of TKI with the aberrant BCR-ABL. The most prevalently observed mutation in CML patients resistant to imatinib is T315I. This mutation has isoleucine instead of threonine at the 315th amino acid in the BCR-ABL protein. Alterations in critical contact points due to amino acid substitutions increase the failure of TKI affinity to the target site. Amino acid substitutions at 7 residues result in mutations such as G250E, M244V, M351T, E255K/V, F359V, Y253F/H, and T315I. To date, over 90 point substitution mutations in BCR-ABL kinase domain have been distinguished in drug resistant CML patients. There are 4 regions that are essential for high frequency binding of imatinib (P-loop, SH-3, SH-2, and A-loop). The P-loop is responsible for phosphate binding and mutations in this site were frequently observed in 43% of patients who were generally in the acute and blast crisis phases. The P-loop mutations E255K and Y253F increase the probability of transformation depending on BCR-ABL kinase activity [24].

BCR-ABL independent resistance is due to LysM-containing receptor-like kinase (Lyn kinase) overexpression [25] and activation [26], increased levels of imatinib efflux transporters [e.g., ATP-binding cassette, sub-family B multidrug resistance (MDR)] [27], and the multidrug resistant protein 1 (MDA-1) [28].

### 3.3. Second Generation TKIs

To overcome intolerance and imatinib resistance in CML patients, development of second-generation TKIs such as dasatinib, bosutinib, and nilotinib was unfolded [29]. Various clinical studies like Dasatinib versus Imatinib Study in Treatment of Naive CML-Chronic Phase-Patients (DASISION) [30], Evaluating Nilotinib Efficacy and Safety in Clinical Trials-Newly Diagnosed Patients (ENESTnd) [31], and Bosutinib Efficacy and Safety in Newly Diagnosed CML (BELA) [32] trials showed efficacy and superiority of second-generation TKIs over first-generation TKI [33].

Dasatinib, a thiazolecarboxamide (Figure 2) binds to the BCR-ABL's ATP binding site with more compatibility than imatinib [34]. Dasatinib is capable of binding to both the open and closed conformations of BCR-ABL whereas imatinib binds only to the closed conformation of the BCR-ABL [35]. Dasatinib was affirmed by the US FDA in 2007 [36]. Interestingly, dasatinib not only inhibits BCR-ABL and but also inhibits kinases like Src kinases (Src, Lck, and YES), c-Kit, PDGFR, and ephrin-A receptor [37].

Nilotinib, an aminopyrimidine (Figure 2) structurally related to imatinib, binds exclusively to the closed conformation of BCR-ABL. Nilotinib has more affinity in binding to the ATP-binding site of BCR-ABL than imatinib [38]. Nilotinib was approved in 2007 by US FDA as a subsequent treatment preference in CML patients in chronic phase, following imatinib. Nilotinib is similar to imatinib in inhibiting PDGFR, Arg, and Kit except Src kinases [39].

According to the instructions of the European Society for Medical Oncology and National Comprehensive Cancer Network Guidelines in Oncology (NCCN) we recommend imatinib, dasatinib, or nilotinib as first line therapy for lately found CML patients in chronic phase [5].

Bosutinib is a 4-anilino-3-quinoline carbonitrile (Figure 2) that inhibits both ABL and Src kinases [40]. Bosutinib is recommended for imatinib resistance CML patients as well as in recently diagnosed CML patients in chronic phase [41]. Importantly, low doses of bosutinib inhibit BCR-ABL when compared to imatinib. Bosutinib is effective against most Gleevac resistant mutations except T315I and V299L substitutions [42].

### 3.4. Third-Generation TKI

BCR-ABL-T315I is the leading mutation that accounts for resistance to cytotoxic drugs, first- and second-generation TKIs. To combat imatinib resistance in CML patients, third-generation TKIs were developed.

Ponatinib (AP24534) is a third-generation TKI (Figure 2) that inhibits both wild type and BCR-ABL harboring mutations such as M244V, G250E, Q252H, Y253F/H, E255K/V, F317L, M351T, and F359V. Ponatinib is developed to inhibit exclusively T315I mutation of BCR-ABL [43].

## 4. *In Vitro* CML Cell Line Model

Currently, more than 40 CML derived cell lines have been documented. Of these, K562 is a BCR-ABL positive CML cell line that was established from 53-year-old woman with CML in blast crisis stage [44]. After 176 passages in 3.5 years, K562 cell line still has active CML cells in blast crisis stage with Ph [45]. In fact, CML induction in mice is carried out by injecting K562 cells into their tail vein. K562 is well reliable BCR-ABL positive CML model to test the efficacy of a desired drug [46].

## 5. Heat Shock Proteins: An Overview

Initially discovered in* Drosophila* by Ritossa in 1996 [47], heat shock proteins (Hsps) function as molecular chaperones facilitating proper folding and maturation of nascent polypeptides [48]. Hsps protect against protein aggregation in cytosol [49]. Hsps are highly conserved and universally expressed in all species from bacteria to plants and animals [50]. These are produced by cells under stressful conditions such as heat, hence the name heat shock proteins. Later, Hsps were found to be rapidly explicated in response to stress conditions such as hypoxia, ischemia, hyperoxia, anoxia, exposure to UV light, nutrient deficiencies (e.g., glucose deprivation), and heavy metals [51]. Heat shock transcriptional factors (HSFs) are protein family that supervises the transcription of Hsp genes [52]. In mammals, major HSFs include HSF1, HSF2, and HSF4. Of these, HSF1 is the main regulator of Hsps production [53].

Under normal physiological conditions, HSF1 remains as a monomer and is tightly bound to Hsp90 [54]. In response to stress, because of increased denatured and improperly folded proteins in the cell, HSF1 dissociates from the Hsp90-HSF1 complex. Released HSF1 from the cytosol translocates into the nucleus and binds to heat shock response element (HSRE) in DNA and induces the production of Hsps. Hsps prevent protein denaturation in cells facing stress conditions [55].

### 5.1. Classification of Hsps

Based on their molecular weight, Hsps have been classified into five major classes. They are small Hsps, Hsp60, Hsp70, Hsp90, and Hsp100. Out of these, Hsp90 is a major cytosolic protein that constitutes about 1-2% of total cytosolic protein [56]. It exhibits four isoforms which include Hsp90*α*, Hsp90*β*, glucose-regulated protein-94 (GRP-94), and TNF receptor associated protein-1 (TRAP-1) [57]. Of these, Hsp90*α* is the major isoform [58].

### 5.2. Hsp90 Structure

Hsp90 exists as a homodimer. It consists of three major regions—a 25 kDa N-terminal domain, 40 kDa middle domain, and 12 kDa C-terminal domain [59]. In addition, a charged linker region between the N-terminal and the middle domain is located. The N-terminal domain has an ATP binding site and shows homology to other ATPase/kinases like gyrase, histidine kinase, and mutL super family [60]. Various client proteins bind to the middle domain of Hsp90 [61]. C-Domain has an alternate ATP binding site. At the end of C-terminal, a tetratricopeptide repeat (TPR) motif, which has a preserved MEEVD pentapeptide intended for Hsp90 interaction with its co-chaperons, is present [62]. Hsp90 mediated protein folding is an ATP dependent process. Various cochaperons like cell division control protein 37 (cdc37), Hsp organizing protein (Hop), activator of Hsp90 ATPase (Aha1), and p23 and FK506-binding protein 51/52 (FKBP51/52) aid Hsp90 in the appropriate folding of client proteins [63].

## 6. Hsp90 and Cancer

**Figure 3:**
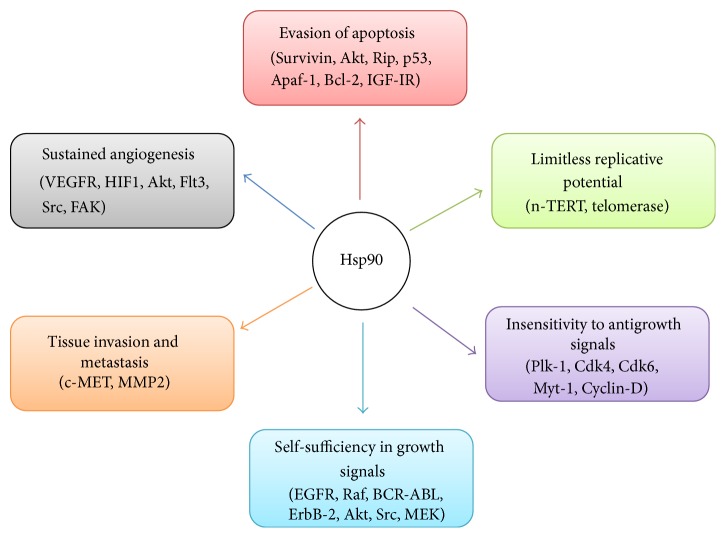
Relationship between Hsp90 and various client proteins resulting in cancer cell survival, progression, invasion, and metastasis.

When compared to normal cells, cancer cells are under physiological stress like hypoxia, ischemia, and nutrient deficiencies (e.g., glucose deprivation). Hence, production of Hsps is ten times higher in tumor cells. Many proteins/factors associated with the six hallmarks of cancer (Figure 3) such as Akt, Src, Flt3, cdk4, cdk6, telomerase, MEK, Raf, HIF1, and BCR-ABL are client proteins of Hsp90 [64].

Moreover, it is now well documented that Hsp90 is requisite for stabilization and proper functioning of these multiple mutated, cancerous proteins that assist cancer cell growth, survival, and malignancy [65]. More than 200 client proteins including hormone receptors, structural proteins [66], tyrosine kinases, and transcription factors [67] require Hsp90 for proper functioning and facilitating growth and survival [68]. Cancer cells exploit Hsp90 to back these activated oncoproteins that are foremost for oncogenic alteration. In this manner, Hsp90 assist cancer cells to survive in an inhospitable environment [69]. A specific role of Hsp90 in maintenance of tumor cell is to inhibit apoptosis [70].

## 7. Hsp90 and CML

**Figure 4:**
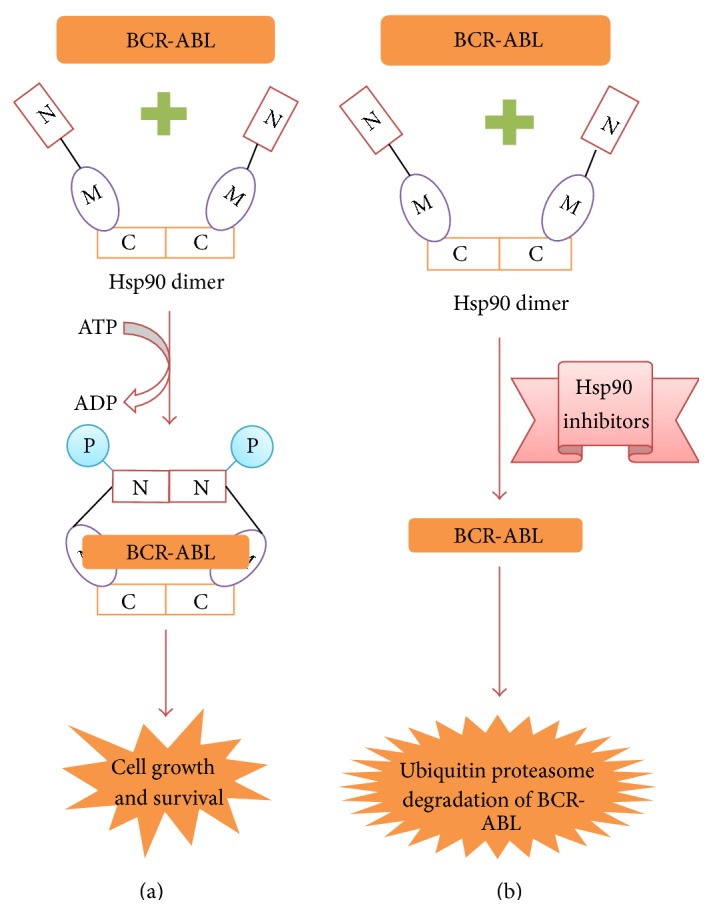
BCR-ABL functioning in the presence and absence of Hsp90 inhibitors. (a) In CML cells, Hsp90 levels are elevated. Hence oncoprotein BCR-ABL binds to Hsp90 for stabilization and maturation. Hence, stabilized BCR-ABL then activates many signaling pathways leading to CML cells survival, progression, and malignancy. (b) Blocking Hsp90 chaperone activity by employing Hsp90 inhibitors results in BCR-ABL degradation via ubiquitin proteasome pathway.

In normal cells Hsp90 levels are low, whereas in CML cells Hsp90 levels are elevated. Hence, elevated Hsp90 levels could serve as prognostic marker in CML [71]. Increased Hsp90 protect fusion oncoprotein BCR-ABL by inhibiting its degradation (Figure 4(a)). In this way, Hsp90 prevent CML cells from apoptosis and promote cell survival and progression. Blocking the binding of BCR-ABL to Hsp90, through Hsp90 inhibitors, causes BCR-ABL degradation via ubiquitin proteasomal pathway (Figure 4(b)) (Table 2).

### 7.1. Hsp90 Inhibitors from Microbial Origin

#### 7.1.1. Benzoquinone Ansamycin Derivatives

**Figure 5:**
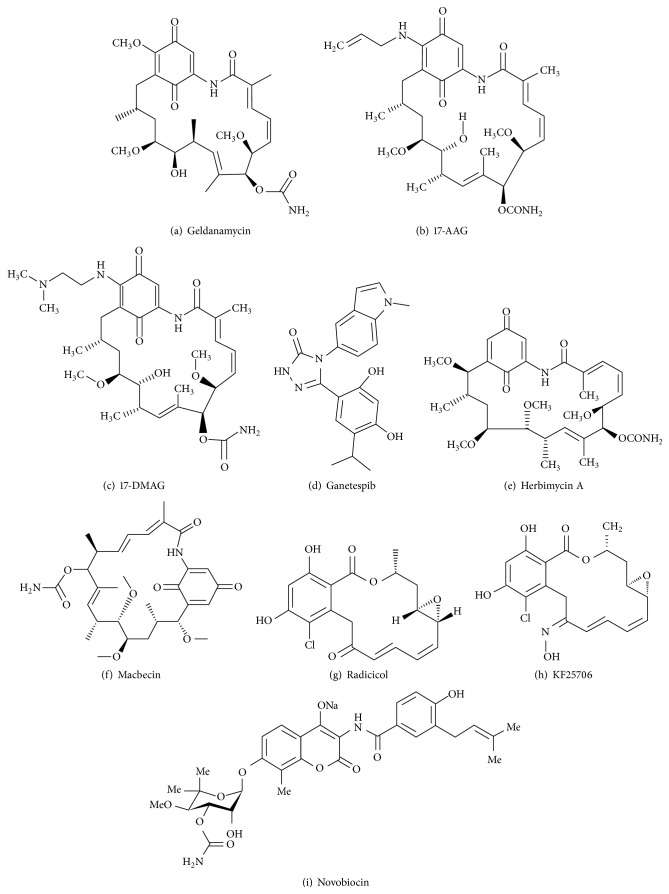
Hsp90 inhibitors of microbial origin.

Geldanamycin (GDA) was isolated from* Streptomyces hygroscopicus* [72]. GDA is a benzoquinone ansamycin antibiotic that binds to the N-terminal domain of Hsp90 and inhibits its activity (Figure 5). GDA is poorly soluble in water and highly hepatotoxic due to the presence of methyl group at C-17. Analogues of GDA, 17-allylamino-17-demethoxygeldanamycin (17AAG) or tanespimycin and 17-dimethylaminoethylamino-17-demethoxygeldanamycin (17DMAG) or alvespimycin (Figure 5) were consequently synthesized. 17AAG is more soluble and showed more potent activity than GDA [73]. Both GDA and 17AAG lowered BCR-ABL levels and lead CML cells to apoptosis. In addition to BCR-ABL, GDA lowered cellular levels of c-Raf and Akt [74]. 17DMAG showed higher potency, more bioavailability, and less toxicity compared to 17AAG and has entered into clinical trials [75]. 17DMAG along with PD184352, a mitogen protein kinase inhibitor, downregulated ERK1/2 and Bcl-xL levels and caused apoptosis in CML cells sensitive as well as resistant to imatinib [76]. The synergistic effect of LBH589, a histone deacetylase inhibitor, and 17AAG downregulated p-Flt3, p-Akt, p-ERK1/2, and BCR-ABL levels in CML cells [77]. The combinational treatment of 17AAG and histone deacetylase inhibitor, suberoylanilide hydroxamic acid, caused mitochondrial damage, activated caspases 3 and 9, and induced apoptosis in CML cells. It also lowered Mcl-1, Raf-1, p27, p34, and p-ERK1/2 levels [78].

Ganetespib (STA-9090) is a non-geldanamycin resorcinol containing triazolone (Figure 5) that is presently in phase II clinical trials for the treatment of solid tumor and hematological malignancy [79]. Ganetespib inhibits Hsp90 activity by blocking the coupling of p23 cochaperone to Hsp90. Ganetespib (IC_50_ is 2–30 nM) is more potent than 17AAG (IC_50_ is 20–3500 nM) and caused cell death in non-small cell lung cancer (NSCLC) models [80]. Ganetespib showed potent activity against primary AML cells and also in combination with cytarabine caused significant cell death [81].

Herbimycin A is a 19-membered macrocyclic lactam (Figure 5) isolated from* Streptomyces hygroscopicus* [82]. Herbimycin A exhibits herbicidal, antifungal, antiangiogenic, and antitumor activities. Herbimycin A modulates Hsp90 thus interrupting it from coupling to its client proteins [83]. The synergistic effect of herbimycin A and TKI made CML cells more susceptible to apoptosis [84].

Macbecin I and Macbecin II (Figure 5) isolated from* Nocardia* species belong to benzoquinone ansamycin group [85]. Macbecin sticks to the ATP binding site of N-terminal of Hsp90. Macbecin is more stable and soluble than GDA. Macbecin I exhibited cytotoxic and antitumor activities against leukemia P388, melanoma B16, and Ehrlich carcinoma in mice [86].

#### 7.1.2. Radicicol

Radicicol is a 14-membered macrolide (Figure 5) isolated from* Diheterospora chlamydosporia* and* Monosporium bonorden*. Like GDA, radicicol binds to the ATP binding site located at the N-terminus of Hsp90 [87]. Radicicol lacks* in vivo* anticancer activity as it is converted to inactive metabolites* in vivo*. To overcome this, oxime derivatives of radicicol such as KF25706, KF29518, and KF58333 (Figure 5) were synthesized [88]. Among these, KF25706 destabilizes interaction between Hsp90 and its associated molecules [89]. Radicicol decreased phosphorylated Raf-1 and BCR-ABL protein levels in CML cells. Radicicol also caused BCR-ABL inactivation via Hsp90 inhibition in K562 cells [90].

#### 7.1.3. Coumermycin Family

Novobiocin (NB), Coumermycin A1 (Figure 5), and Clorobiocin belong to the coumermycin antibiotic family. They were isolated from* Streptomyces spheroids*. NB binds avidly to the C-terminal of Hsp90 and inhibits its activity [91]. NB reduced p-Akt, p-ERK, and p-BCR-ABL levels in CML cells. NB also decreased the coupling of BCR-ABL to Hsp90 and caused BCR-ABL degradation via proteasome-ubiquitin pathway in K562 cells. Moreover NB not only inhibited proliferations but also lowered levels of BCR-ABL in K562 cells resistant to imatinib [92].

### 7.2. Hsp90 Inhibitors from Plant Origin

#### 7.2.1. Epigallocatechin-3-gallate (EGCG)

**Figure 6:**
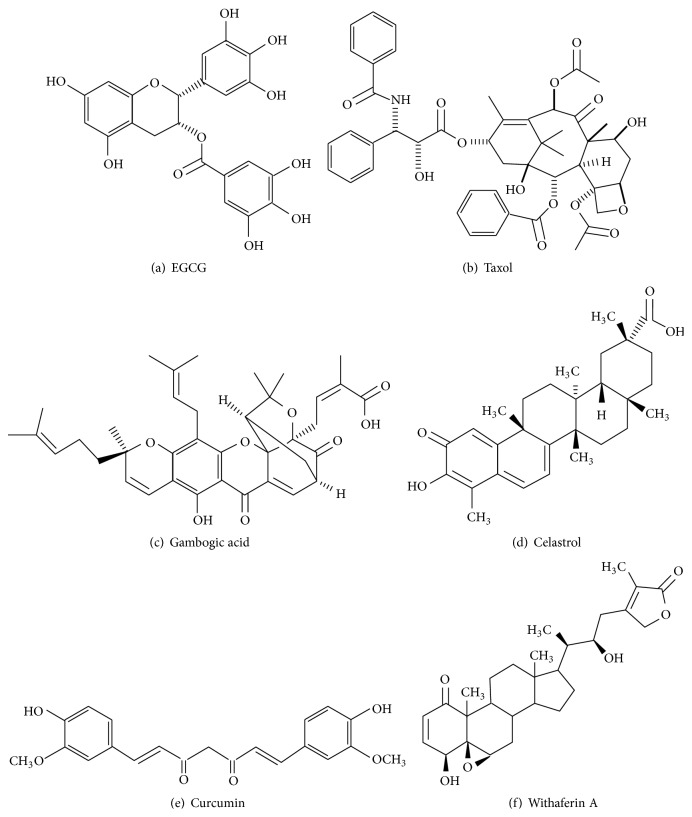
Hsp90 inhibitors of plant origin.

EGCG is a polyphenol compound (Figure 6) isolated from green tea* Camellia sinensis* [93]. EGCG is a C-terminus inhibitor and suppresses Hsp90 activity [94, 95]. In combination with ponatinib, EGCG induced significant apoptosis in CML cells. CyclinD1 and CDC25A levels were downregulated by this treatment in CML cells [96].

#### 7.2.2. Taxol

Taxol (Figure 6), isolated from* Taxus baccata* L. [97], agent stabilizes microtubule formation and induces apoptosis via mitotic catastrophe [98]. Taxol inhibits Hsp90 activity [99, 100]. Synergistic effect of paclitaxel and bortezomib, a proteasome inhibitor, reduced the autophosphorylated levels of BCR-ABL. Moreover, this combinational treatment attenuated BCR-ABL downstream signaling pathways, decreasing phosphorylated STAT3, STAT5, CRKL, and Lyn levels. It also activates caspases 3, 8, and 9 causing apoptosis of CML cells via poly(ADP-Ribose) polymerase (PARP) cleavage [101].

#### 7.2.3. Gambogic Acid (GA)

GA is a main component of (Figure 6) Chinese plant,* Garcinia hanburyi* (gamboge) [102]. GA binds to N-terminus of Hsp90 and inhibits its activity. GA was approved by China FDA and entered into phase-II clinical trials. GA deregulates the expression of Src-3, Akt, and VEGFR2, resulting in the apoptosis of tumor cells [103]. GA not only induced apoptosis but also reduced cell proliferation in CML cells. GA lowered BCR-ABL levels in wild and resistant CML cells* in vivo*. GA decreased the levels of p-STAT5, p-CRKL, p-ERK1/2, and p-Akt [104].

#### 7.2.4. Celastrol

Celastrol is a quinone methide triterpene (Figure 6) isolated from the poisonous plant* Tripterygium wilfordii* Hook F (Thunder God Vine). Celastrol is used in the medication against inflammation, asthma, and allergic conditions [105]. Celastrol disrupts the binding of Cdc37 to Hsp90, thus inhibiting its activity [106]. Importantly, celastrol triggers the degradation of BCR-ABL and Flt3 [107]. Both* in vitro* and* in vivo* studies showed that celastrol decreased phosphorylated BCR-ABL, Akt, Erk1/2, and STAT5 protein levels. Celastrol also lowered antiapoptotic (Bcl-xL, Mcl-1, and survivin) protein levels and induced apoptosis in CML cells harboring T135I mutation [108].

#### 7.2.5. Curcumin

Curcumin, isolated from Curcuma plant species (Figure 6) like* Curcuma aromatica*,* Curcuma longa*, and* Curcuma phaeocaulis*, exhibits antioxidant, anti-inflammatory, and antitumor activities. Several lines of studies demonstrated the antileukemic activity on ALL cells both* in vitro* and* in vivo*. Curcumin inhibits ABL, STAT, Akt, and mTOR signaling pathways causing apoptosis of CML cells [109]. Curcumin inhibits Hsp90 activity [110]. C817, a novel derivative of curcumin, inhibits both wild and mutant ABL kinase activities (Q252H, Y253F, and T315I). In addition, C817 downregulates phosphorylated BCR-ABL, CRKL, and STAT5 protein levels [111].

#### 7.2.6. Withaferin A

Withaferin A is a steroidal lactone (Figure 6) isolated from the Indian medicinal plant, Ashwagandha* (Withania somnifera)* [112]. Withaferin A interferes with Hsp90-Cdc37 chaperone complex by regulating leucine rich repeat kinase 2 (LRRK2) levels [113]. Withaferin A reduced the levels of NF*κ*B and potently inhibited the growth of human and murine B cell lymphoma cell lines [114]. Withaferin A induced caspase activation and caused apoptosis in doxorubicin-sensitive K562 and doxorubicin-resistant K562/Adr cells. Moreover, it downregulated Bcl-2, Bim, p-Bad, and cytoskeletal tubulin protein levels. Hence, Withaferin A overcomes the MDR caused by the overexpression of P-glycoprotein (P-gp) in K562 levels with attenuated apoptosis [115].

### 7.3. Hsp90 Inhibitors from Coral Origin

#### 7.3.1. 5-Episinuleptolide Acetate (5EPA)

**Figure 7:**
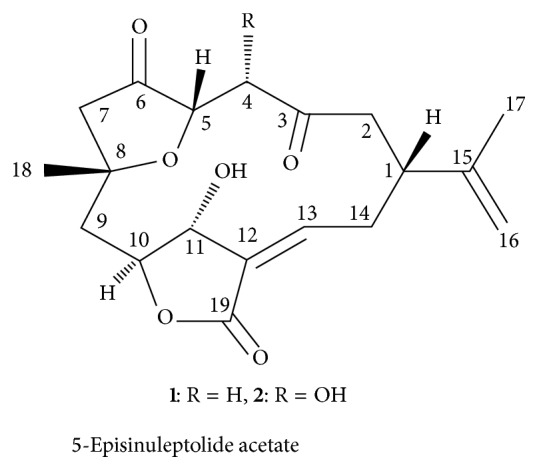
Hsp90 inhibitors of coral origin.

5EPA is a macrocyclic 3(2H)-furanone-based norcembranoidal diterpene (Figure 7) isolated from the formosan soft coral* Sinularia* species. 5EPA exhibits antiproliferative activity against K562, HL60, and Molt4 cancer cell lines. 5EPA also lowered c-ABL, Akt, and NF*κ*B levels in CML cells. 5EPA induced apoptosis in leukemic cells via Hsp90 inhibition [116].

## 8. Evaluation of Hsp90 Function or Hsp90 Inhibition

Hsp90 function or inhibition could be evaluated by the following assays. They are as follows.

### 8.1. Coupled Enzyme Assay

**Figure 8:**
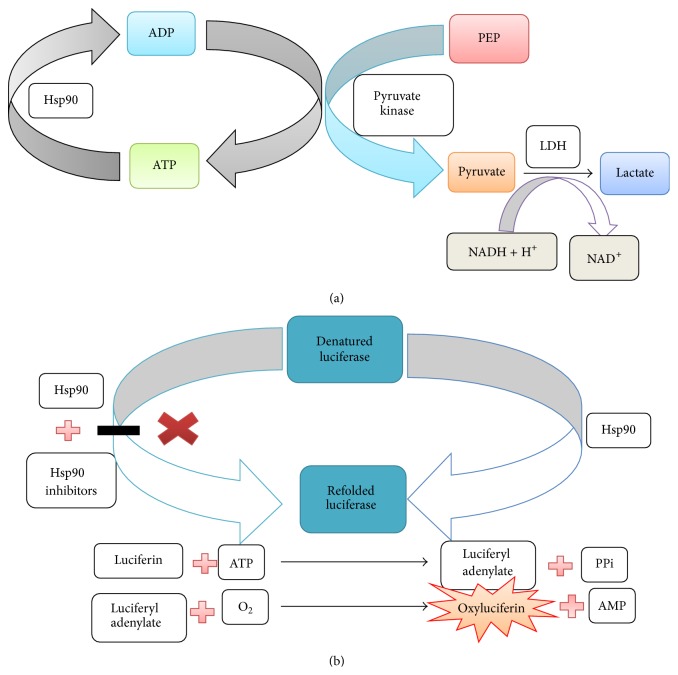
(a) Coupled enzyme assay. (b) Luciferase refolding assay.

Hsp90 requires ATP for its molecular chaperone activity [63]. The ADP generated by Hsp90 is coupled with phosphoenol pyruvate to generate ATP and pyruvate catalyzed by pyruvate kinase enzyme. Then, pyruvate is converted into lactate by lactate dehydogenase enzyme with the expenditure of NADH. This assay is done to assess the ATPase activity of Hsp90. In this assay for every ADP generated by Hsp90, one NADH is consumed (Figure 8(a)). Low NADH levels lead to the decrease in UV absorbance at 340 nm [117].

### 8.2. Malachite-Green Assay

This assay is also designed to evaluate the ATPase activity of Hsp90 [63]. The cationic dye malachite-green along with the phosphomolybdate reacts with inorganic phosphate released to give a blue color having absorption maxima at 610 nm [118, 119].

### 8.3. Hsp90 Inhibition Using Western Blotting

Hsp90 inhibition leads to the depletion of Hsp90 levels and its associated molecules in cells. The decreased Hsp90 levels could be observed by employing western blotting and densitometry studies [117].

### 8.4. Luciferase Refolding Assay

Luciferase is a bioluminescent photoprotein enzyme derived from firefly* Photinus pyralis.* Luciferase catalyzes the reaction of luciferin with ATP forming luciferyl adenylate, which in turn reacts with oxygen giving oxyluciferin. This reaction emits light [120]. In this assay the capability of a drug to inhibit Hsp90 is evaluated (Figure 8(b)). Denatured luciferase along with rabbit reticulocyte lysate (rich source of Hsp90 and its cochaperons) is incubated. The desired drug molecule is also incubated along with the above two. If the drug molecule is effective, it will inhibit the refolding of luciferase by Hsp90 [121].

## 9. Hsp90 Inhibitors in Clinical Trials

Of the several Hsp90 inhibitors identified, 17-AAG (NCT00100997) is currently under phase I clinical trial sponsored by Jonsson Comprehensive Cancer Center collaborated with National Cancer Institute (NCI) [122]. Efforts are undergoing to determine the side effects and best dose of 17-AAG in treating patients with CML in chronic phase that did not respond to imatinib mesylate. Ganetespib (NCT00964873) is in phase I clinical study to assess the safety and efficacy of once-weekly administered STA-9090 in patients with AML, ALL, and blast-phase CML. This study is sponsored by Synta Pharmaceuticals Corporation [123]. Paclitaxel (NCT00003230) is under Phase I/II trials to study the effectiveness in treating patients with refractory or recurrent acute leukemia or CML. This work is sponsored by Swiss Group for Clinical Cancer Research [124].

## 10. Conclusions

CML is a haematological malignancy that arises due to chromosomal translocation resulting in the presence of Ph chromosome. Initially, TKIs were designed to compete with the ATP binding site of BCR-ABL. TKIs effectively inhibited wild BCR-ABL; however mutations in BCR-ABL and overexpression following treatment decreased their potency. Specifically, T315I mutation displayed resistance to both first- and second-generation TKIs. Eventually, ponatinib was developed to inhibit mutant T315I and was approved by the US FDA but was withdrawn due to toxicity.

Presently, there is a need for alternative strategy to develop new BCR-ABL inhibitors. Hsp90 is upregulated in CML cells for rapid proliferation, survival, and progression towards malignancy. Several Hsp90 inhibitors have been shown to inhibit and degrade BCR-ABL.* In vitro* studies unveiled that Hsp90 inhibitors alone or in combination with other inhibitors downregulate BCR-ABL levels in wild-type and TKI-resistant CML cells. Thus, suppressing the BCR-ABL protein levels through Hsp90 inhibition renders an attractive alternate strategy to combat CML.

## Figures and Tables

**Table 1:** Anti-CML TKIs.

TKIs	Compound name	Previous name	Company	Trade name	Approval by US, FDA	Dosage
First-generation TKI	Imatinib	STI571, CPG57148B	Novartis	Gleevac or Glivec	2001	CML-CP-400 mg once daily

Second-generation TKIs	Nilotinib	AMN107	Novartis	Tasigna	2006	CML- CP/AP-400 mg twice daily
	Dasatinib	BMS354825	Bristol-Myers Squibb	Sprycel	2007	CML-CP-100 mg once daily
	Bosutinib	SKI606	Pfizer	Bosulif	2012	500 mg once daily

Third-generation TKI	Ponatinib	AP24534	Ariad	Iclusig	2013	45 mg once daily

^*∗*^CP: chronic phase; AP: accelerated phase.

**Table 2:** BCR-ABL signaling pathways affected by Hsp90 inhibitors.

S. number	Name of the compound	Isolated from	Nature of origin	Mechanism of inhibition of Hsp90	Signaling protein downregulated by Hsp90 inhibitors	References
1	Geldanamycin	*Streptomyces hygroscopicus*	Bacterial	Binds to N-terminal domain of Hsp90	↓ c-Raf, ↓ Akt, ↓ BCR- ABL	[74]

2	Radicicol	*Diheterospora chlamydosporia *and *Monosporium bonorden*	Fungal	Binds to N-terminal domain of Hsp90	↓ p-Raf1, ↓ p-BCR- ABL	[90]

3	Novobiocin	*Streptomyces spheroids*	Bacterial	Binds to C-terminal domain of Hsp90	↓ p-Akt, ↓ p-ERK & ↓ p-BCR- ABL	[91, 92]

4	Epigallocatechin-3-gallate	*Camellia sinensis*	Plant	Binds to C-terminal domain of Hsp90	↓ CyclinD1, ↓ CDC25A	[96]

5	Taxol	*Taxus baccata *L.	Plant	NYK	↓ pSTAT3, ↓ pSTAT5, ↓ p-CRKL, ↓ p-Lyn	[101]

6	Gambogic acid	*Garcinia hanburyi*	Plant	Binds to N-terminal domain of Hsp90	↓ p-BCR- ABL ↓ pSTAT5, ↓ p-CRKL, ↓ pERK1/2, ↓ p-Akt & ↓ p-BCR- ABL	[103, 104]

7	Celastrol	*Tripterygium wilfordii *Hook F	Plant	Disrupts binding of Cdc37 to Hsp90	↓ pSTAT5, ↓ p-CRKL, ↓ pERK1/2, ↓ p-Akt, ↓ p-BCR- ABL, ↓ Bcl-xL, ↓ Mcl-1, ↓ survivin	[107, 108]

8	Curcumin	*Curcuma aromatica, Curcuma longa*, and *Curcuma phaeocaulis*	Plant	NYK	↓ BCR- ABL, ↓ CRKL, ↓ STAT5	[110, 111]

9	Withaferin A	*Withania somnifera*	Plant	Disrupts the binding of Cdc37 to Hsp90	↓ NF*κ*B, ↓ Bcl-2, ↓ Bim, ↓ p-Bad	[114, 115]

10	5-Episinuleptolide acetate	*Sinularia *species	Coral	NYK	↓ c-ABL, ↓ Akt, ↓ NF*κ*B	[116]

^*∗*^NYK: not yet known.

## Abbreviations

CLL: Chronic lymphoid leukemia
ALL: Acute lymphoid leukemia
CML: Chronic myeloid leukemia
AML: Acute myeloid leukemia
Ph: Philadelphia chromosome
ABL: c-Abelson
BCR: Break point cluster region
MAPK: Mitogen-activated protein kinase
STAT: Signal transducers and activator of transcription
PI3K: Phosphoinositide 3-kinases
BUS: Busulfan
HU: Hydroxyurea
IFN-*α*: Interferon-*α*
TKI: Tyrosine kinase inhibitors
IRIS: International Randomized Study of Interferon and STI571
FDA: Food and Drug Administration
Lyn kinase: LysM-containing receptor-like kinase
MDR: Multidrug resistance
MDA-1: Multidrug resistant protein 1
DASISION: Dasatinib versus Imatinib Study In Treatment of Naive CML-CP patients
ENESTnd: Evaluating Nilotinib Efficacy and Safety in Clinical Trials-Newly Diagnosed Patients
BELA: Bosutinib Efficacy and Safety in Newly Diagnosed CML
NCCN: National Comprehensive Cancer Network Guidelines in Oncology
Hsps: Heat shock proteins
HSFs: Heat shock transcriptional factors
HSRE: Heat shock Response Element
TPR: Tetratricopeptide repeat
GRP-94: Glucose-regulated protein-94
TRAP-1: TNF receptor associated protein-1
cdc37: Cell division control protein 37
Hop: Hsp organizing protein
Aha1: Activator of Hsp90 ATPase
FKBP51/52: FK506-binding protein 51/52
GDA: Geldanamycin
17AAG: 17-Allylamino-17-demethoxygeldanamycin
17DMAG: 17-Dimethylaminoethylamino-17-demethoxygeldanamycin
NSCLC: Non-small cell lung cancer
NB: Novobiocin
EGCG: Epigallocatechin-3-gallate
PARP: Poly(ADP-Ribose) polymerase
GA: Gambogic acid
LRRK2: Leucine rich repeat kinase 2
5EPA: 5-Episinuleptolide acetate
P-gp: P-Glycoprotein
NCI: National Cancer Institute.
